# 1-Allyl-5-nitro-1*H*-benzimidazol-2(3*H*)-one

**DOI:** 10.1107/S1600536813004790

**Published:** 2013-02-23

**Authors:** Younès Ouzidan, Youssef Kandri Rodi, Adiba Kandri Rodi, El Mokhtar Essassi, Mohamed Saadi, Lahcen El Ammari

**Affiliations:** a Laboratoire de Chimie Organique Appliquée, Université Sidi Mohamed Ben Abdallah, Faculté des Sciences et Techniques, Route d’immouzzer, BP 2202 Fès, Morocco; b Laboratoire de Chimie Organique Hétérocyclique URAC21, Faculté des Sciences, Université Mohammed V-Agdal, Avenue Ibn Battouta, BP 1014, Rabat, Morocco; c INANOTECH (Institute of Nanomaterials and Nanotechnology), MAScIR, Av. de l’Armée Royale, Rabat, Morocco; d Laboratoire de Chimie du Solide Appliquée, Faculté des Sciences, Université Mohammed V-Agdal, Avenue Ibn Battouta, BP 1014, Rabat, Morocco

## Abstract

The benzimidazolone residue in the title mol­ecule, C_10_H_9_N_3_O_3_, is almost planar, with the largest deviation from the mean plane being 0.016 (2) Å for the C atom linked to the nitro group. This plane is nearly perpendicular to the 1-allyl chain as indicated by the C—N—C—C torsion angle of 90.9 (3)°. The fused-ring system makes a dihedral angle of 5.6 (3)° with the nitro group, leading to a synperiplanar conformation. In the crystal, zigzag supra­molecular chains are formed along the *a* axis by N—H⋯O hydrogen bonds.

## Related literature

For pharmacological and biochemical properties of benz­imid­azoles and derivatives, see: Al Muhaimeed (1997 ▶); Scott *et al.* (2002 ▶); Nakano *et al.* (2000 ▶); Zhu *et al.* (2000 ▶); Zarrinmayeh *et al.* (1998 ▶). For related structures, see: Ouzidan *et al.* (2011*a* ▶,*b* ▶).
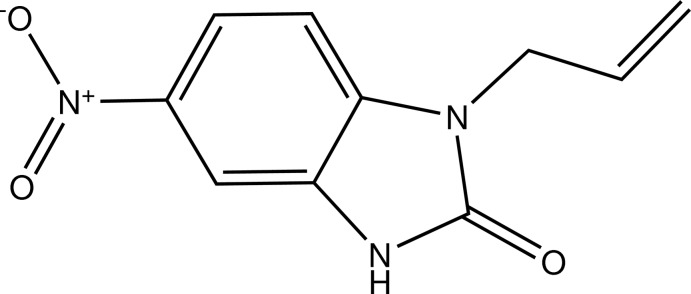


## Experimental

### 

#### Crystal data

C_10_H_9_N_3_O_3_
*M* *_r_* = 219.20
Orthorhombic, 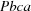

*a* = 8.3246 (3) Å
*b* = 14.9567 (6) Å
*c* = 16.4461 (7) Å
*V* = 2047.68 (14) Å^3^
*Z* = 8
Mo *K*α radiation
μ = 0.11 mm^−1^
*T* = 296 K
0.46 × 0.31 × 0.18 mm

#### Data collection

Bruker X8 APEXII area-detector diffractometer
12145 measured reflections
1940 independent reflections
1483 reflections with *I* > 2σ(*I*)
*R*_int_ = 0.030

#### Refinement

*R*[*F* ^2^ > 2σ(*F* ^2^)] = 0.057
*wR*(*F* ^2^) = 0.168
*S* = 1.05
1940 reflections
145 parameters
H-atom parameters constrained
Δρ_max_ = 0.60 e Å^−3^
Δρ_min_ = −0.38 e Å^−3^

### 

Data collection: *APEX2* (Bruker, 2009 ▶); cell refinement: *SAINT* (Bruker, 2009 ▶); data reduction: *SAINT*; program(s) used to solve structure: *SHELXS97* (Sheldrick, 2008 ▶); program(s) used to refine structure: *SHELXL97* (Sheldrick, 2008 ▶); molecular graphics: *ORTEP-3 for Windows* (Farrugia, 2012 ▶); software used to prepare material for publication: *PLATON* (Spek, 2009 ▶) and *publCIF* (Westrip, 2010 ▶).

## Supplementary Material

Crystal structure: contains datablock(s) I, global. DOI: 10.1107/S1600536813004790/tk5200sup1.cif

Structure factors: contains datablock(s) I. DOI: 10.1107/S1600536813004790/tk5200Isup2.hkl

Supplementary material file. DOI: 10.1107/S1600536813004790/tk5200Isup3.cml

Additional supplementary materials:  crystallographic information; 3D view; checkCIF report

## Figures and Tables

**Table 1:** Hydrogen-bond geometry (Å, °)

*D*—H⋯*A*	*D*—H	H⋯*A*	*D*⋯*A*	*D*—H⋯*A*
N2—H2*N*⋯O3^i^	0.86	1.96	2.801 (3)	164

Symmetry code: (i) 
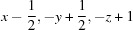
.

**Fig. 1.**
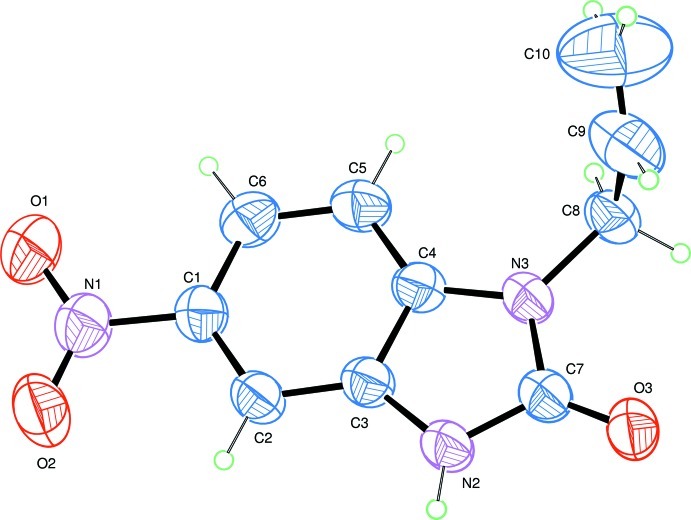
Molecular structure of the title compound with the atom-labelling scheme. Displacement ellipsoids are drawn at the 50% probability level. H atoms are represented as small circles.

**Fig. 2.**
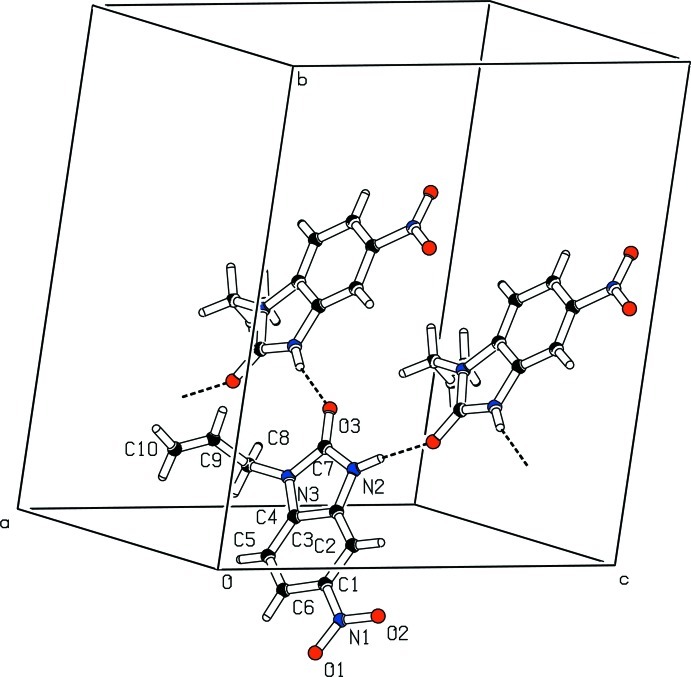
Molecule and its symmetry partner linked by N2—H2n···O3 hydrogen bonds. Symmetry codes: (i) 1/2 - x, 1 - y, 1/2 + z.

## supplementary crystallographic information

### Comment

Benzimidazoles and their derivatives exhibit a number of important pharmacological properties, such as anti-histaminic (Al Muhaimeed, 1997) anti-ulcerative (Scott *et al.*, 2002) and anti-allergic (Nakano *et al.*, 2000). In addition, benzimidazole derivatives are effective against the human cytomegalovirus (HCMV) (Zhu *et al.*, 2000) and are also efficient selective neuropeptide Y Y1 receptor antagonists (Zarrinmayeh *et al.*, 1998).

As a continuation of our research work devoted to the development of substituted benzimidazol-2-one derivatives (Ouzidan *et al.*, 2011*a*, 2011*b*), we report in this paper the synthesis of a new benzimidazol-2-one derivative by action of allyl-bromide with 5-nitro-1*H*-benzo[*d*]imidazol-2(3*H*)one using similar conditions as employed in earlier studies.

The two fused five- and six-membered rings in the molecule of the title compound, C_10_H_9_N_3_O_3_, are approximately planar, the largest deviation from the mean plane being -0.016 (2) A° at C1 (Fig. 1). The dihedral angle between the benzimidazolone mean plane and the 1-allyl chain (C4—N3—C8—C9) is 90.9 (3)°. The fused-ring system makes a dihedral angle of 5.6 (3)° with the nitro group, leading to a syn-periplanar conformation. In the crystal, each molecule is linked to symmetry equivalents by N2—H2n···O3 hydrogen bonds, Table 2, forming a supramolecular zigzag chain running along the *a* axis, as shown in Fig. 2.

### Experimental

To 5-Nitro-1*H*-benzo[*d*]imidazol-2(3*H*)-one (0.2 g, 1.11 mmol), potassium carbonate (0.3 g, 2.23 mmol), and tetra-*n*-butylammonium bromide (0.04 g, 0.11 mmol) in DMF (15 ml) was added allyl bromide (0.11 ml, 1.35 mmol). Stirring was continued at room temperature for 6 h. The salts were removed by filtration and the filtrate concentrated under reduced pressure. The residue was separated by chromatography on a column of silica gel with ethyl acetate/hexane (1/2) as eluent. Crystals were isolated when the solvent was allowed to evaporate.

### Refinement

H atoms were located in a difference map and treated as riding with N—H = 0.86 Å, C—H = 0.93 Å (aromatic) and C—H = 0.97 Å (methylene), and with *U*_iso_(H) = 1.2 *U*_eq_(parent atom). Disorder is noted in the allyl group as evidence by the shorter than normal C9═C10 bond length of 1.177 (5) Å. Attempts to resolve this disorder for this room temperature data set were not successful.

### Crystal data

**Table:**

C_10_H_9_N_3_O_3_	*F*(000) = 912
*M**_r_* = 219.20	*D*_x_ = 1.422 Mg m^−^^3^
Orthorhombic, *P**b**c**a*	Mo *K*α radiation, λ = 0.71073 Å
Hall symbol: -P 2ac 2ab	Cell parameters from 1940 reflections
*a* = 8.3246 (3) Å	θ = 3.0–25.7°
*b* = 14.9567 (6) Å	µ = 0.11 mm^−^^1^
*c* = 16.4461 (7) Å	*T* = 296 K
*V* = 2047.68 (14) Å^3^	Block, colourless
*Z* = 8	0.46 × 0.31 × 0.18 mm

### Data collection

**Table:**

Bruker X8 APEXII area-detector diffractometer	1483 reflections with *I* > 2σ(*I*)
Radiation source: fine-focus sealed tube	*R*_int_ = 0.030
Graphite monochromator	θ_max_ = 25.7°, θ_min_ = 3.0°
φ and ω scans	*h* = −10→5
12145 measured reflections	*k* = −17→18
1940 independent reflections	*l* = −20→17

### Refinement

**Table:**

Refinement on *F*^2^	Primary atom site location: structure-invariant direct methods
Least-squares matrix: full	Secondary atom site location: difference Fourier map
*R*[*F*^2^ > 2σ(*F*^2^)] = 0.057	Hydrogen site location: inferred from neighbouring sites
*wR*(*F*^2^) = 0.168	H-atom parameters constrained
*S* = 1.05	*w* = 1/[σ^2^(*F*_o_^2^) + (0.0827*P*)^2^ + 1.3415*P*] where *P* = (*F*_o_^2^ + 2*F*_c_^2^)/3
1940 reflections	(Δ/σ)_max_ < 0.001
145 parameters	Δρ_max_ = 0.60 e Å^−^^3^
0 restraints	Δρ_min_ = −0.38 e Å^−^^3^

### Special details

**Table:**

Geometry. All e.s.d.'s (except the e.s.d. in the dihedral angle between two l.s. planes) are estimated using the full covariance matrix. The cell e.s.d.'s are taken into account individually in the estimation of e.s.d.'s in distances, angles and torsion angles; correlations between e.s.d.'s in cell parameters are only used when they are defined by crystal symmetry. An approximate (isotropic) treatment of cell e.s.d.'s is used for estimating e.s.d.'s involving l.s. planes.
Refinement. Refinement of *F*^2^ against all reflections. The weighted *R*-factor *wR* and goodness of fit *S* are based on *F*^2^, conventional *R*-factors *R* are based on *F*, with *F* set to zero for negative *F*^2^. The threshold expression of *F*^2^ > σ(*F*^2^) is used only for calculating *R*-factors(gt) *etc*. and is not relevant to the choice of reflections for refinement. *R*-factors based on *F*^2^ are statistically about twice as large as those based on *F*, and *R*- factors based on all data will be even larger.

### Fractional atomic coordinates and isotropic or equivalent isotropic displacement parameters (Å^2^)

**Table:**

	*x*	*y*	*z*	*U*_iso_*/*U*_eq_	
C1	0.1801 (3)	−0.05403 (17)	0.37164 (15)	0.0479 (6)	
C2	0.1529 (3)	0.02563 (16)	0.41211 (15)	0.0469 (6)	
H2	0.0502	0.0439	0.4269	0.056*	
C3	0.2866 (3)	0.07620 (16)	0.42913 (14)	0.0433 (6)	
C4	0.4416 (3)	0.04786 (16)	0.40696 (14)	0.0432 (6)	
C5	0.4658 (3)	−0.03268 (18)	0.36771 (16)	0.0517 (6)	
H5	0.5685	−0.0516	0.3538	0.062*	
C6	0.3328 (3)	−0.08415 (18)	0.34984 (16)	0.0532 (6)	
H6	0.3449	−0.1387	0.3234	0.064*	
C7	0.4612 (3)	0.18307 (17)	0.46777 (15)	0.0470 (6)	
C8	0.7198 (3)	0.11726 (18)	0.41762 (15)	0.0505 (6)	
H8A	0.7613	0.0567	0.4152	0.061*	
H8B	0.7714	0.1474	0.4628	0.061*	
C9	0.7608 (4)	0.1651 (3)	0.3401 (2)	0.0817 (10)	
H9	0.7305	0.2249	0.3380	0.098*	
C10	0.8257 (7)	0.1378 (4)	0.2815 (3)	0.1325 (19)	
H10A	0.8593	0.0786	0.2794	0.159*	
H10B	0.8434	0.1755	0.2375	0.159*	
N1	0.0402 (3)	−0.10842 (16)	0.34996 (15)	0.0589 (6)	
N2	0.3035 (2)	0.15796 (14)	0.46649 (13)	0.0506 (6)	
H2N	0.2257	0.1889	0.4863	0.061*	
N3	0.5459 (2)	0.11428 (13)	0.43179 (12)	0.0455 (5)	
O1	0.0603 (3)	−0.17533 (15)	0.30908 (16)	0.0871 (8)	
O2	−0.0927 (2)	−0.08426 (14)	0.37286 (15)	0.0746 (7)	
O3	0.5177 (2)	0.25247 (13)	0.49549 (13)	0.0607 (5)	

### Atomic displacement parameters (Å^2^)

**Table:**

	*U*^11^	*U*^22^	*U*^33^	*U*^12^	*U*^13^	*U*^23^
C1	0.0473 (13)	0.0443 (14)	0.0521 (13)	−0.0050 (11)	0.0021 (10)	−0.0018 (11)
C2	0.0375 (12)	0.0459 (14)	0.0573 (14)	0.0000 (10)	0.0064 (10)	−0.0019 (11)
C3	0.0391 (12)	0.0427 (13)	0.0479 (13)	0.0012 (10)	0.0048 (9)	−0.0019 (10)
C4	0.0395 (12)	0.0459 (13)	0.0441 (12)	0.0020 (10)	0.0034 (9)	−0.0012 (10)
C5	0.0453 (13)	0.0507 (15)	0.0592 (15)	0.0083 (11)	0.0064 (11)	−0.0064 (12)
C6	0.0573 (15)	0.0438 (14)	0.0584 (15)	0.0040 (11)	0.0039 (12)	−0.0081 (11)
C7	0.0381 (12)	0.0489 (14)	0.0541 (14)	−0.0002 (10)	0.0035 (10)	−0.0042 (11)
C8	0.0331 (12)	0.0633 (16)	0.0551 (14)	0.0048 (11)	0.0010 (10)	−0.0028 (12)
C9	0.0555 (17)	0.110 (3)	0.080 (2)	0.0014 (18)	0.0127 (16)	0.012 (2)
C10	0.181 (5)	0.122 (4)	0.094 (3)	0.021 (4)	0.050 (3)	0.018 (3)
N1	0.0592 (14)	0.0470 (13)	0.0704 (15)	−0.0068 (10)	0.0009 (11)	−0.0055 (11)
N2	0.0353 (10)	0.0469 (12)	0.0695 (13)	−0.0001 (8)	0.0088 (9)	−0.0147 (10)
N3	0.0332 (10)	0.0492 (12)	0.0542 (12)	−0.0002 (8)	0.0036 (8)	−0.0051 (9)
O1	0.0777 (15)	0.0703 (15)	0.1133 (18)	−0.0149 (11)	0.0075 (13)	−0.0401 (13)
O2	0.0483 (11)	0.0586 (13)	0.1169 (18)	−0.0083 (9)	0.0026 (11)	−0.0109 (11)
O3	0.0435 (9)	0.0561 (11)	0.0825 (13)	−0.0072 (8)	0.0022 (9)	−0.0194 (9)

### Geometric parameters (Å, º)

**Table:**

C1—C2	1.383 (3)	C7—N2	1.365 (3)
C1—C6	1.396 (4)	C7—N3	1.381 (3)
C1—N1	1.465 (3)	C8—N3	1.467 (3)
C2—C3	1.374 (3)	C8—C9	1.501 (4)
C2—H2	0.9300	C8—H8A	0.9700
C3—N2	1.376 (3)	C8—H8B	0.9700
C3—C4	1.407 (3)	C9—C10	1.177 (5)
C4—N3	1.381 (3)	C9—H9	0.9300
C4—C5	1.381 (3)	C10—H10A	0.9300
C5—C6	1.380 (4)	C10—H10B	0.9300
C5—H5	0.9300	N1—O1	1.217 (3)
C6—H6	0.9300	N1—O2	1.223 (3)
C7—O3	1.228 (3)	N2—H2N	0.8600
			
C2—C1—C6	123.4 (2)	N3—C8—C9	111.9 (2)
C2—C1—N1	117.7 (2)	N3—C8—H8A	109.2
C6—C1—N1	118.8 (2)	C9—C8—H8A	109.2
C3—C2—C1	116.1 (2)	N3—C8—H8B	109.2
C3—C2—H2	122.0	C9—C8—H8B	109.2
C1—C2—H2	122.0	H8A—C8—H8B	107.9
C2—C3—N2	131.5 (2)	C10—C9—C8	129.3 (4)
C2—C3—C4	121.6 (2)	C10—C9—H9	115.3
N2—C3—C4	106.84 (19)	C8—C9—H9	115.3
N3—C4—C5	132.4 (2)	C9—C10—H10A	120.0
N3—C4—C3	106.5 (2)	C9—C10—H10B	120.0
C5—C4—C3	121.2 (2)	H10A—C10—H10B	120.0
C6—C5—C4	118.0 (2)	O1—N1—O2	122.5 (2)
C6—C5—H5	121.0	O1—N1—C1	118.8 (2)
C4—C5—H5	121.0	O2—N1—C1	118.7 (2)
C5—C6—C1	119.7 (2)	C7—N2—C3	110.48 (19)
C5—C6—H6	120.1	C7—N2—H2N	124.8
C1—C6—H6	120.1	C3—N2—H2N	124.8
O3—C7—N2	127.4 (2)	C7—N3—C4	109.99 (19)
O3—C7—N3	126.4 (2)	C7—N3—C8	123.3 (2)
N2—C7—N3	106.2 (2)	C4—N3—C8	126.5 (2)
			
C6—C1—C2—C3	1.2 (4)	C2—C1—N1—O2	−4.6 (4)
N1—C1—C2—C3	−178.0 (2)	C6—C1—N1—O2	176.2 (3)
C1—C2—C3—N2	178.7 (2)	O3—C7—N2—C3	179.0 (2)
C1—C2—C3—C4	−0.4 (4)	N3—C7—N2—C3	−1.3 (3)
C2—C3—C4—N3	179.1 (2)	C2—C3—N2—C7	−178.3 (3)
N2—C3—C4—N3	−0.2 (3)	C4—C3—N2—C7	0.9 (3)
C2—C3—C4—C5	−0.6 (4)	O3—C7—N3—C4	−179.1 (2)
N2—C3—C4—C5	−179.9 (2)	N2—C7—N3—C4	1.2 (3)
N3—C4—C5—C6	−178.8 (2)	O3—C7—N3—C8	−3.4 (4)
C3—C4—C5—C6	0.9 (4)	N2—C7—N3—C8	176.9 (2)
C4—C5—C6—C1	−0.1 (4)	C5—C4—N3—C7	179.1 (3)
C2—C1—C6—C5	−1.0 (4)	C3—C4—N3—C7	−0.6 (3)
N1—C1—C6—C5	178.2 (2)	C5—C4—N3—C8	3.5 (4)
N3—C8—C9—C10	−117.9 (5)	C3—C4—N3—C8	−176.1 (2)
C2—C1—N1—O1	174.6 (3)	C9—C8—N3—C7	−84.0 (3)
C6—C1—N1—O1	−4.6 (4)	C9—C8—N3—C4	90.9 (3)

### Hydrogen-bond geometry (Å, º)

**Table:**

*D*—H···*A*	*D*—H	H···*A*	*D*···*A*	*D*—H···*A*
N2—H2*N*···O3^i^	0.86	1.96	2.801 (3)	164

Symmetry code: (i) *x*−1/2, −*y*+1/2, −*z*+1.
